# Genetic, epigenetic, and post‐transcriptional basis of divergent tissue regenerative capacities among vertebrates

**DOI:** 10.1002/ggn2.10042

**Published:** 2021-05-17

**Authors:** Sheamin Khyeam, Sukjun Lee, Guo N. Huang

**Affiliations:** ^1^ Cardiovascular Research Institute and Department of Physiology University of California San Francisco California USA; ^2^ Eli and Edythe Broad Center for Regeneration Medicine and Stem Cell Research University of California San Francisco California USA

**Keywords:** appendage, heart, regeneration, spinal cord

## Abstract

Regeneration is widespread across the animal kingdom but varies vastly across phylogeny and even ontogeny. Adult mammalian regeneration in most organs and appendages is limited, while vertebrates such as zebrafish and salamanders are able to regenerate various organs and body parts. Here, we focus on the regeneration of appendages, spinal cord, and heart—organs and body parts that are highly regenerative among fish and amphibian species but limited in adult mammals. We then describe potential genetic, epigenetic, and post‐transcriptional similarities among these different forms of regeneration across vertebrates and discuss several theories for diminished regenerative capacity throughout evolution.

## Graphical abstract

Regeneration is widespread across the animal kingdom but varies vastly across phylogeny and even ontogeny. Adult mammalian regeneration in most organs and appendages is limited, while vertebrates such as zebrafish and salamanders are able to regenerate various organs and body parts. In this review, we describe potential genetic, epigenetic, and post‐transcriptional similarities among heart, appendage, and spinal cord regeneration across vertebrates and discuss several theories for diminished regenerative capacity throughout evolution.

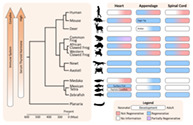

## 1 INTRODUCTION TO REGENERATION IN THE ANIMAL KINGDOM

Regenerative capacity varies widely in the animal kingdom. Invertebrate species such as planarians are champions of regeneration, capable of regenerating the entire organism.^1^, ^2^ Within vertebrate species, those most closely resembling their fossil ancestors—“phylogenetically primitive” organisms such as fish (with zebrafish being a prominent example) and urodele amphibians—are capable of regenerating the limb, fin, spinal cord, heart, and other organs. Meanwhile, adult mammalian species are largely limited to regenerating specific tissues such as the skin, liver, and skeletal muscle and not body parts such as the heart, spinal cord, and limbs.^3^, ^4^, ^5^ Strikingly, neonatal mammals retain a lot of the regenerative capacity observed in fish and amphibian species. Whether this regenerative trait is a unique regeneration response, a modified injury response, or a combination of both remains unclear. The subsequent critical question is whether regeneration is an ancestral trait lost through evolution or a novel trait gained. Despite evidence supporting both theories, the ancestral trait theory has been gaining popularity with increasing evidence of conserved genetic and molecular pathways governing regeneration across phylogeny.^6^

**FIGURE 1:**
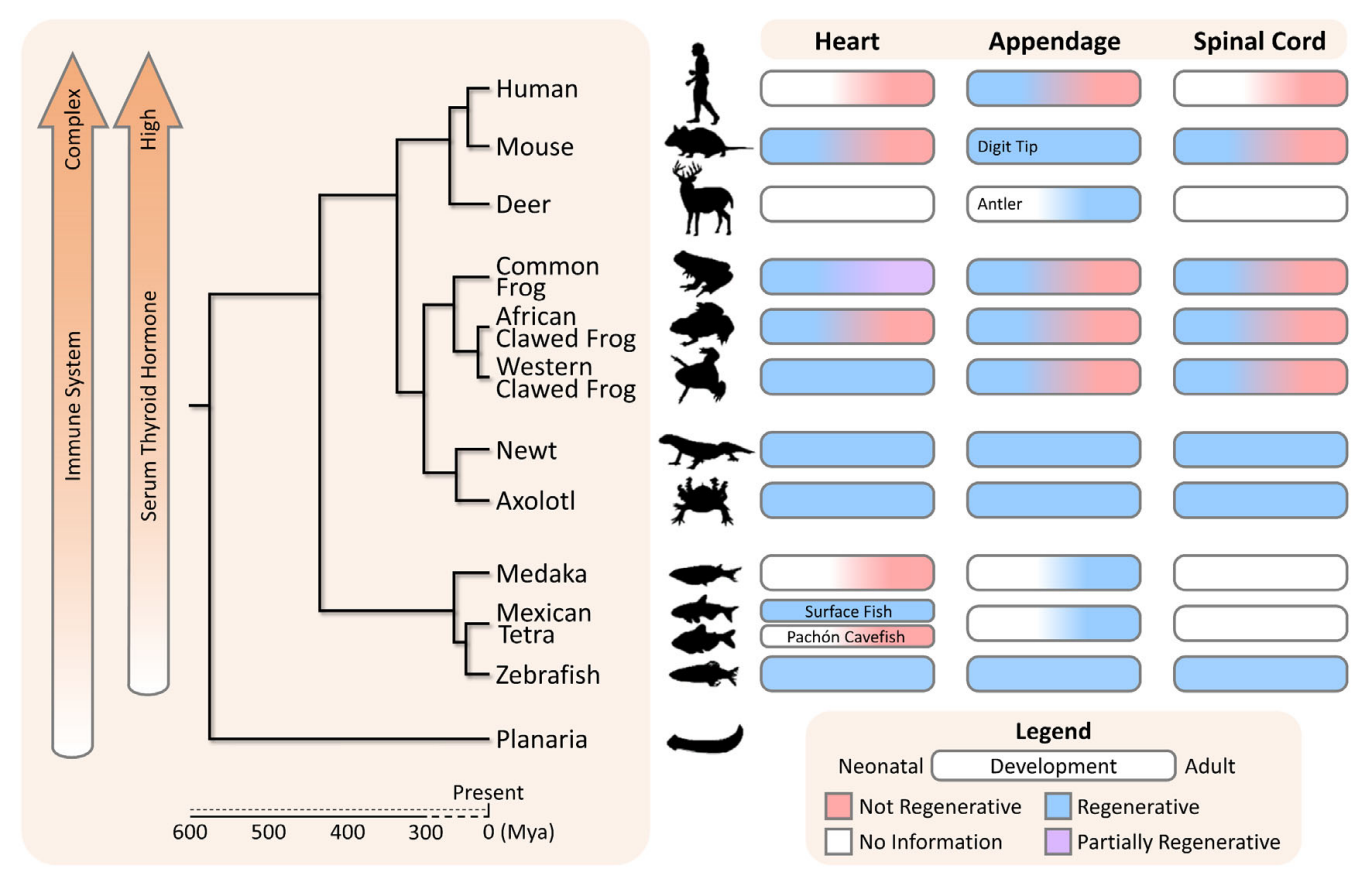
Phylogenetic distribution of species demonstrating varied heart, appendage, and spinal cord regenerative capacities across development

**FIGURE 2:**
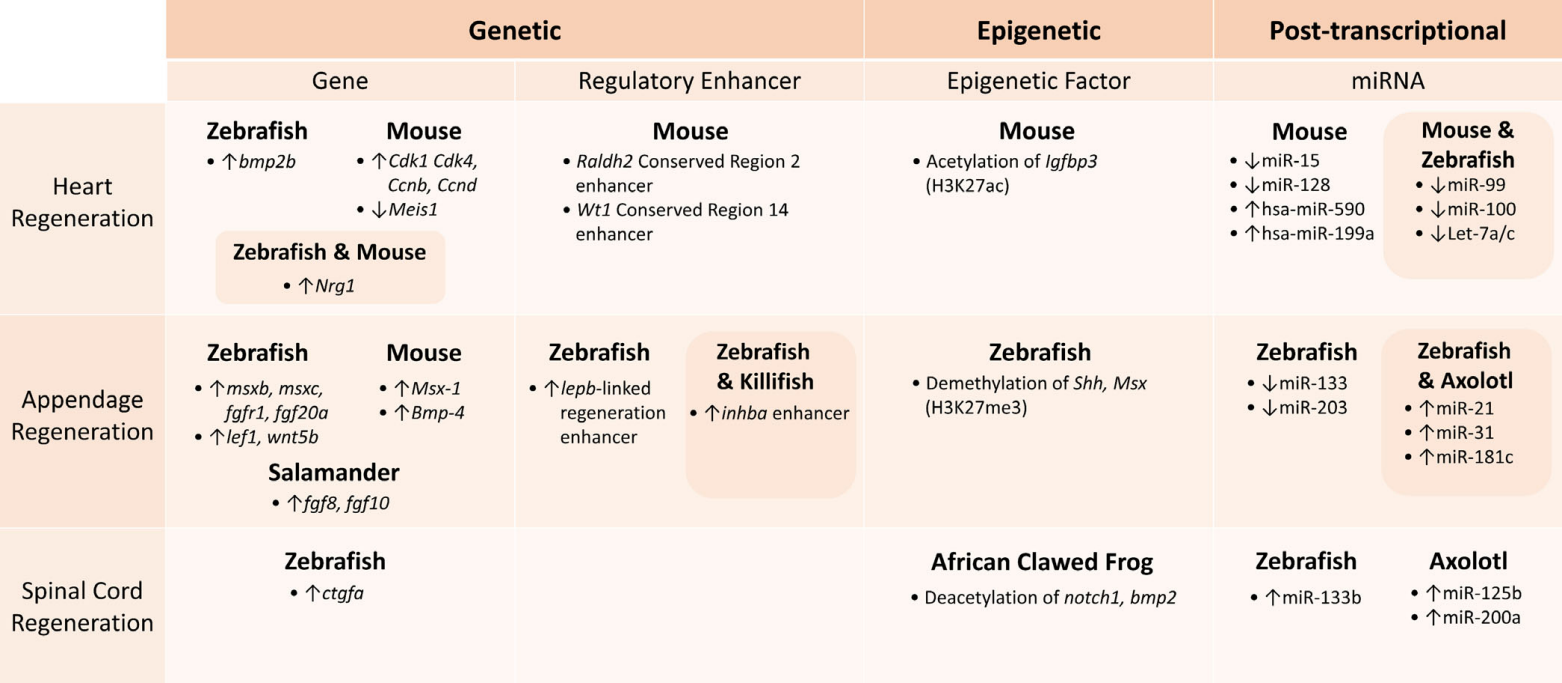
Table of essential genetic, epigenetic, and post‐transcriptional regulators of regeneration categorized by heart, appendage, or spinal cord regeneration. Arrows indicate whether overexpression or inhibition promotes regeneration

This review will first briefly introduce regeneration in invertebrate species, then focus on examining the diverse regenerative capacities of appendage, spinal cord, and heart in key model organisms including zebrafish (*Danio rerio*), axolotl salamanders (*Ambystoma mexicanum*), frogs (*Rana* spp., *Xenopus* spp.), and mice (laboratory strains of *Mus musculus* unless otherwise identified). We hope to highlight some genetic and epigenetic commonalities of regeneration across vertebrate species and address a few theories seeking to explain the general decline in regenerative capacity accompanying evolutionary development (Figures 1 and 2).

### 1.1 Regeneration in invertebrates

Unlike complex vertebrates, most invertebrates demonstrate a robust ability to regenerate.^7^ For instance, the planarian flatworm (*Schmidtea mediterranea*) is prized for its astonishing regenerative potential.^1^ *Planaria maculata* fully restore their bodies when left with a mere 1/279th fraction of the original body.^8^ Throughout development, planaria rely on neoblast stem cells to regenerate tissues; upon injury, positional control genes, such as *notum* and *wnt1*, are activated to re‐establish positional information and serve as guides for progenitor cell differentiation.^9^ On the other hand, insects demonstrate limited regenerative capacity—possibly due to their complex, derived morphogenesis—and are an anomaly amongst invertebrates.^10^ For example, *Drosophila melanogaster* imaginal discs, which develop into limbs, appendages, and other parts, are highly regenerative during early larval stages but lose the ability to regenerate in larval development as they differentiate exoskeletal cuticle.^11^ Still, many invertebrates of both the protostome and deuterostome lineages, such as flatworms and echinoderms, retain high regenerative potential.^7^

### 1.2 Appendage regeneration in vertebrates

Contemporary vertebrate appendage regeneration models follow a similar order of events: epithelial closure of the wound site, establishment of a wound epithelium, dedifferentiation or activation of cells leading to epimorphic blastema formation, and proliferation and redifferentiation of progenitor cells to regenerate various appendage cell types in a lineage‐restricted manner (or what appears to be lineage‐restricted in species thoroughly investigated thus far).^12^ The model is not all‐encompassing: morphologically simpler vertebrates can fully regenerate appendages, while more complex vertebrates generally possess extremely limited appendage regeneration.

Regeneration of the teleost fin was first documented by French naturalist Broussonet, then further explored by Thomas Hunt Morgan.^13^, ^14^, ^15^, ^16^ To date, the signaling pathways essential for zebrafish fin regeneration include many that are necessary for vertebrate embryonic development. Specifically, Wnt/β‐catenin, RA, IGF, and FGF signaling pathways drive blastema formation and expression of *fgf20a*, essential for initiating fin regeneration and subsequent proliferative outgrowth.^14^, ^17^, ^18^

Axolotl salamanders (*Ambystoma mexicanum*) are among the oldest studied regenerative models with the first evidence of whole adult tail and limb regeneration documented by Spallanzani.^19^ Molecular pathways similar to those in zebrafish fin regeneration also drive salamander limb regeneration. For instance, FGF signaling is required for blastema formation, with *fgf8* and *fgf10* expression essential for successful completion.^20^ In addition, Wnt/β‐catenin signaling is necessary during early stages of limb regeneration but not after blastema formation.^21^, ^22^

Although complete limb regeneration is not observed in mammals, neonatal mice, adult mice, and humans can regenerate digit tips amputated through the distal region of the terminal phalanx.^23^, ^24^, ^25^ Amputations transecting more proximal regions, however, result in regenerative failure and scarring instead.^26^ Mammalian digit tip regeneration correlates with regions that express *Msx‐1* and *Msx‐1‐*mutant mice cannot regenerate the digit tip.^27^, ^28^ However, BMP‐4 can rescue this phenotype, suggesting the importance of the BMP/Msx signaling pathway in digit tip regeneration.^28^

Antlers in all Cervidae and female reindeer (*Rangifer tarandus*) are the only mammalian appendages that can fully regenerate, not only once, but many times throughout the host's lifetime. Scholars still debate the classification of this form of annual tissue replacement since seasonal hormonal changes, rather than injuries, prompt antler regeneration. Regardless, deer antlers are used as a model to understand complete mammalian appendage regeneration being a unique example of stem cell‐based epimorphic regeneration that also depends on blastema‐based appendage regeneration pathways.^29^, ^30^, ^31^, ^32^

### 1.3 Spinal cord regeneration in vertebrates

Spinal cord injuries in adult mammals generate permanent glial scars and lead to motor and sensory impairment.^33^, ^34^ Studying organisms capable of spinal cord regeneration can provide insights into various injury responses permitting pro‐regenerative environments. Here, we will discuss zebrafish and salamanders, species regenerative throughout their lifetimes, as well as frogs and mice, species only regenerative soon after birth.

Upon injury, the adult zebrafish spinal cord triggers proliferation and differentiation of resident neural progenitor cells called ependymoradial glial cells.^35^ Spinal cord regeneration reuses major developmental pathways including Notch, Hh, Wnt, and FGF signaling.^36^, ^37^ FGF signaling in particular induces glial cell bridge formation, which provides a scaffold for regenerating axonal growth. Moreover, *connective tissue growth factor a* (*ctgfa*) is necessary and sufficient to initiate glial cell bridging and regeneration.^38^, ^39^

Spallanzani first described axolotl salamander spinal cord regeneration in 1768; subsequent studies of the field appeared in the mid‐20th century.^40^, ^41^, ^42^, ^43^, ^44^ After tail amputation, salamanders can regenerate their spinal cord through ependymoradial glial cell proliferation and differentiation, similar to zebrafish.^45^, ^46^, ^47^ Radial glial cells in axolotl tail regeneration can switch lineages to generate surrounding muscle and cartilage tissue in addition to regenerating a functional spinal cord.^48^ This phenomenon contrasts the emerging theme that transdifferentiation is not a major mechanism governing limb and fin regeneration.

*Xenopus*, more phylogenetically complex than fish and salamanders, are able to regenerate their spinal cords as larvae but not post‐metamorphosis.^49^ Unlike the axolotl, each main tissue group in the regenerated tail—notochord, spinal cord, and myofibers—arises from its respective remaining tissue in a lineage‐restricted fashion (again, given current limitations on genetic tools).^50^, ^51^ Regeneration‐organizing cells are present and essential during tail development and regeneration.^52^ These cells express multiple regeneration‐supportive genes (*Wnt5a*, *Fgf10*, *Fgf20*, and *Msx*) and simultaneously upregulate ligand expression for common pro‐regenerative signaling pathways.^52^ Although similar cell types have yet to be discovered in other regeneration models, regeneration‐organizing cells offer a fresh perspective to current known regenerative mechanisms.

Recent studies have revealed the capability of neonatal mice to heal, scar‐free, after spinal cord injuries. This discovery dispels the long‐standing theory that mammals are incapable of such regeneration.^53^ Following injury, microglia transiently secrete fibronectin and binding proteins to form bridges across the severed spinal cord (like that in zebrafish) and express peptidase inhibitors to help resolve inflammation.^53^ Future studies can help identify key genetic and molecular factors regulating pro‐regenerative traits of neonatal, but not adult, microglia.

### 1.4 Cardiac regeneration in vertebrates

Similar to spinal cord regeneration, adult mammalian hearts have limited regenerative potential compared to certain fish and amphibians. The general consensus within available models across phylogeny is that cardiac regeneration depends on the dedifferentiation and proliferation of pre‐existing cardiomyocytes.^54^, ^55^, ^56^, ^57^, ^58^, ^59^

Zebrafish—and other teleost fish including Giant Danio (*Devario aequipinnatus*)^60^ and goldfish (*Carassius auratus*)^61^ but not medaka (*Oryzias latipes*)^62^—retain robust regenerative capacity throughout life; adult zebrafish can regenerate their heart without scarring within 2 months post‐injury after 20% ventricular amputation.^63^ Although zebrafish reuse many developmental signaling pathways seen in other models of regeneration, Jak/Stat, BMP, and Notch pathways are regeneration‐specific and not essential for cardiac development.^64^ All three signaling pathways regulate cardiomyocyte proliferation after injury; *bmp2b* overexpression, in particular, is sufficient to enhance cardiomyocyte dedifferentiation and proliferation.^65^

Mexican tetra (*Astyanax mexicanus*) are teleost fish whose surface populations can regenerate the heart. Groups of Mexican tetra diverged into caves and evolved independently 1.5 million years ago, gaining traits not seen in surface fish and losing traits such as their eyes, pigment, and, at least in the Pachón cavefish population, cardiac tissue regeneration.^66^, ^67^ Intra‐species breeding and genetic analyses between these distinct populations provide genomic explanations for differences in cardiac regenerative capacity. *Lrrc10*, a highly conserved gene unique to the heart, is differentially expressed and positively regulates cardiac regeneration in surface fish. Pachón cavefish, which exhibit low endogenous levels of *lrrc10*, and mutant zebrafish with *lrrc10* knocked out, both demonstrate impaired cardiac regeneration.^67^

Initial studies on adult newt (*Notophthalmus viridescens*) cardiac regeneration report incomplete regenerative capacity 30 days post‐injury.^68^, ^69^ Newer studies suggest that adult newts can fully regenerate their hearts 60 days post‐injury, with damaged tissue completely restored structurally and functionally.^57^, ^58^ Molecular pathways governing adult newt cardiac regeneration are not well established due to difficulties in sequencing their complex genome. Transcriptional approaches, however, show strong gene expression changes associated with the extracellular matrix post‐injury, including matrix metalloproteinases, collagen, keratin, and tenascin‐C.^70^, ^71^, ^72^ The extracellular matrix may be important in signaling to the regenerating myocardium. For example, tenascin‐C can promote cell cycle re‐entry of newt cardiomyocytes in vitro.^73^

The adult mammalian heart is mainly thought to be a post‐mitotic organ incapable of regeneration. In contrast, neonatal mice can fully regenerate their hearts without scarring within the first week after birth.^74^ Significant molecular pathways that mediate cardiac regeneration in neonatal mice include Hippo, neuregulin, FGF, and thyroid hormone signaling, each altering cardiomyocyte cell cycle activity.^72^, ^75^, ^76^ Positive cell cycle regulators can promote dedifferentiation and proliferation of post‐mitotic cardiomyocytes, with the combination of cyclin‐dependent kinase 1 (CDK1), CDK4, cyclin B1 (CCNB), and cyclin D1 (CCND) inducing post‐mitotic cell proliferation.^77^ Deletion of *Meis1*—normally required for transcriptional activation of CDK inhibitors—is also sufficient to extend the perinatal cardiomyocyte proliferative window.^78^

### 1.5 Liver Regeneration in Vertebrates

Although more complex vertebrates exhibit limited regenerative potential overall, most, if not all, are capable of liver regeneration.^79^ Upon partial hepatectomy, the liver undergoes both hyperplasia and hypertrophy to regenerate and restore homeostasis.^80^, ^81^ Once signaling pathways targeting hepatocyte growth factor are activated, hepatocytes enter the cell cycle and restore the liver to its normal size.^82^ Rats can regenerate the liver even after 70% partial hepatectomy, while zebrafish are able to after 30% partial hepatectomy.^80^, ^83^ Also, much like other examples of regeneration, zebrafish, rats, and mice utilize BMP, FGF, and Wnt signaling pathways to regenerate the liver.^83^, ^84^

## 2 SIMILARITIES IN REGENERATION ACROSS VERTEBRATES

Despite divergent tissue regeneration capacities observed across the animal kingdom, commonalities exist among appendage, heart, and spinal cord regeneration in fish, amphibians, and mammals. Here, we first highlight how regeneration is generally dependent on nerves, and proceed to discuss conserved genetic, epigenetic, and post‐transcriptional machinery governing regenerative potential.

### 2.1 Nerve dependence

Various organ regeneration in diverse species is nerve‐dependent.^85^ Marcus Singer's neurotrophic hypothesis of regeneration suggests that nerves influence regeneration by producing factors promoting progenitor cell proliferation and differentiation.^86^ These factors are still not clearly characterized today but suggest an evolutionarily conserved role and function of nerves in regeneration across vertebrates.

Seminal work by Tweedy John Todd demonstrates salamander limb regeneration's nerve‐dependent nature.^87^ Limb and fin regeneration occur through blastema formation adjacent to the wound epithelium. If the nerve is severed at the base of the limb prior to or shortly after amputation, the wound epithelium still forms but the apical epithelial cap does not, and limb regeneration fails due to loss of blastema cell proliferation.^85^, ^88^ Proliferation is mediated by the apical epithelial cap dependence on nerves and its interaction with the blastema. In the newt, regenerating axons in the apical epithelial cap synthesize and secrete anterior gradient proteins, which then bind to its receptor Prod1 in the blastema to promote cell cycling.^86^ Local expression of the anterior gradient protein after electroporation into a denervated newt limb blastema is sufficient to induce distal structure regeneration. Moreover, in vitro studies show mesenchymal blastema cells can re‐enter S phase when exposed to recombinant anterior gradient protein.^89^

There are around 100 differentially expressed genes between innervated and denervated Mexican axolotl limbs, half of which are annotated to cell cycle and mitosis‐related ontologies.^90^ Genes encoding cell cycle regulators such as *ccna2* (a positive regulator of cardiac regeneration in mice and pigs^91^, ^92^) and those associated with cell proliferation and differentiation are enriched and only affected during the formation of the blastema following denervation.^90^

In zebrafish fin regeneration, denervation affects mesenchymal cell proliferation, apical epithelial cap formation and signaling, and blastema formation and outgrowth. Genes essential for blastema formation, including *msxb*, *msxc*, *fgfr1*, and *fgf20a*, are significantly altered in denervated fins, suggesting the role of nerves in FGF signal regulation.^88^ In addition, *lef1* and *wnt5b* expression levels are significantly altered in denervated fins, suggesting the nerves' importance in regulating Wnt signaling during regeneration as well.^88^

Although less understood mechanistically, mouse digit tip regeneration is also dependent on innervation. Following amputation, Schwann cell‐derived precursor cells from intact peripheral nerves secrete pro‐proliferative factors that are necessary for digit tip regeneration. These mitogenic factors include oncostatin M and platelet derived growth factor AA, which are sufficient to rescue digit tip regeneration following denervation.^93^

Despite the general consensus that appendage regeneration is dependent on nerves, antler regeneration is in fact nerve independent. Denervation of antlerogenic regions does not affect the pedicles' or first antlers' formation and growth, and denervation of pedicles is not essential for antler regeneration.^94^, ^95^, ^96^ In both cases, however, denervation affects antler size and shape, meaning nerves may help determine antler growth specifically.^94^, ^95^ This may be due to the stem cell‐based, rather than blastema‐based, nature of antler regeneration. Overall, current literature suggests that nerves are crucial for blastema formation in appendage regeneration.

The heart is extensively innervated. Multiple studies show that nerve function is essential for complete cardiac regeneration in adult zebrafish and neonatal mice.^97^, ^98^ Loss of cholinergic signaling in adult zebrafish and neonatal mice leads to reduced cardiomyocyte proliferation, evidenced by reduced expression of cell cycle genes such as *Ccnd2* and *Cdk4* and reduced expression of growth factors *Nrg1* and *Ngf* ^98^. *Nrg1* expression in particular is necessary and sufficient to induce cardiomyocyte proliferation in zebrafish, while administration of Nrg1 and Ngf recombinant proteins can rescue direct mechanical denervation in neonatal mice.^98^, ^99^

### 2.2 Regulatory enhancers

One of the major hypotheses regarding the evolution of regeneration is that regeneration is an innate ability and crucial regenerative genes already exist in most animal genomes; mechanisms to activate them after injury are simply lost. Low regenerative capacity of more complex vertebrates may result from differences in or loss of such regulatory mechanisms.

Enhancer elements are *cis‐*acting DNA regulatory elements that control gene transcription. They can be activated by specific developmental, injury‐related, or regeneration‐specific cues, or a combination thereof. Epicardial enhancer sequences *Raldh2* conserved region 2 and *Wt1* conserved region 14 are activated during development. The transcription factor C/EBP mediates injury‐induced reactivation of these enhancers following myocardial infarction and ischemia reperfusion in adult mice.^100^ This suggests that developmental enhancers can be reused as injury‐responsive enhancers in adult tissue, warranting further investigation of transcriptional mechanisms controlling enhancer element activation as a means of promoting regeneration.

Recently, more regeneration‐associated enhancers have been discovered.^101^, ^102^, ^103^ Transcriptome analysis in the regenerating zebrafish fin and heart reveal *leptin b* (*lepb*) transcription is significantly upregulated during both forms of regeneration. A sequence upstream of *lepb*, described as *lepb*‐linked regeneration enhancer (*LEN*), directs regeneration‐specific gene expression without developmental activity. This enhancer sequence is also separable into regulatory elements with distinct tissue preferences—heart or fin—which are activated by the respective injuries.^103^

An evolutionary emergence or loss of regeneration enhancers through natural selection may explain the wide variance in regenerative capacity across phylogeny. Limited DNA sequence conservation of zebrafish *LEN* is found upstream of the *leptin* gene in mice. Despite this, zebrafish *LEN* is able to direct injury‐dependent gene expression following cardiac injury and digit tip amputation.^103^, ^104^ This suggests that although the regeneration‐specific enhancer was lost in mammals during evolution, regulatory mechanisms to activate zebrafish *LEN* still exist and can be reactivated in the context of an injury. There thus exists the possibility that *LEN* acts more like an injury‐responsive enhancer in mammals.

An outstanding question is whether an enhancer element that is activated following an injury is an injury‐responsive enhancer or a regeneration‐specific enhancer. Moreover, is the regeneration‐specific enhancer function important for tissue regeneration? A recent comprehensive study addresses these questions by comparing zebrafish and killifish fin and heart regeneration.^105^ A set of evolutionarily conserved enhancer regulatory sequences is activated in both species and during both processes. Deletion of a conserved *inhibin beta A* (*inhba*) enhancer element in killifish delays fin regeneration and abrogates heart regeneration. While the predicted zebrafish *inhba* enhancer recapitulates killifish *inhba* enhancer activity and can functionally replace it for effective fin and heart regeneration, the predicted human *inhba* enhancer neither shows the same spatiotemporal activity nor rescues defective fin regeneration in killifish.^105^ Thus, these findings present an example in which both of the following occur simultaneously: enhancer element conservation leading to regenerative competency in simpler vertebrates and enhancer sequence variation resulting in regenerative failure in more complex vertebrates.

### 2.3 Epigenetic modifications

Regardless of the cellular mode of regeneration—self‐renewing stem cells, cell dedifferentiation, or proliferation of pre‐existing cells—extensive epigenetic reprogramming occurs to generate an appropriate regenerative response.^106^ Gene expression is regulated through chromatin structure modifications, *cis*‐regulatory DNA elements, and transcriptional machinery. Understanding evolutionarily conserved gene regulatory mechanisms may, in the future, allow researchers to reactivate dormant regeneration‐associated genes in mammals.

Regenerative processes often reuse genes and pathways involved in development. To ensure that these genes are only activated in a particular spatiotemporal context, epigenetic modifications are required to alter gene accessibility. To illustrate, caudal fin amputation in zebrafish leads to the reactivation of developmental regulators such as *Shh* and *Msx*.^107^, ^108^ Histone demethylases can activate these developmental regulatory genes by removing H3K27me3 marks.^109^ Similarly, *Shh* enhancer methylation status dictates limb regenerative capacity in the *Xenopus*; here, hypomethylation contributes positively to regeneration in tadpoles, but negatively in adults.^110^

Epigenetic modifications also regulate spinal cord regeneration. Histone deacetylases are necessary for both axolotl and *Xenopus* tail regeneration.^111^, ^112^ Histone deacetylase inhibition in the *Xenopus* tail leads to aberrant expression of *notch1* and *bmp2* and regenerative failure.^111^ Likewise, multiple Notch and BMP pathway targets are also dysregulated when a histone deacetylase inhibitor is added to the axolotl tail regeneration model.^112^

*Igf2bp3*, a regulator of IGF2 signaling, is highly expressed in the regenerative neonatal mouse heart and significantly downregulated in the nonregenerating adult heart.^113^ This positive regulator of cardiomyocyte proliferation and cardiac regeneration is initially modified with H3K27ac activators but is then replaced with H3K27me3 silencers during postnatal development.^113^ In zebrafish, H3K27me3 deposition is necessary for silencing structural genes in the heart to complete regeneration.^114^ These findings suggest the critical role epigenetic modifications play in regenerative processes; modifications may both stimulate and stymie regeneration.

### 2.4 miRNA

MicroRNAs (miRNAs) are small noncoding mRNAs that post‐transcriptionally regulate gene expression by degrading their target mRNA and/or inhibiting their translation. They are required for development, stem cell maintenance, injury response, and multiple forms of regeneration.^115^, ^116^, ^117^

miRNAs play a crucial role in zebrafish caudal fin regeneration. FGF‐dependent miR‐133 depletion enhances regeneration by increasing proliferation within the blastema, and miR‐203 downregulation allows for essential *lef1* (a Wnt signaling transcription factor) expression.^118^, ^119^ Furthermore, a cross‐species analysis of miRNA expression accompanying blastema formation in zebrafish caudal fins, bichir pectoral fins, and axolotl forelimbs reveals a shared set of differentially regulated miRNAs. Upregulation of miR‐21, miR‐31 and miR‐181c leads to the downregulation of anti‐proliferative genes such as *pdcd4*, *tgfbr2*, *bcl2l13*, *rgs5* and *chka*, in turn increasing proliferation necessary for blastema formation.^120^

In zebrafish spinal cord regeneration, miR‐133b is upregulated following a complete spinal cord transection and positively regulates regeneration and functional recovery.^121^ In axolotl glial cells, miR‐200a is upregulated and necessary for axonal regrowth and regeneration.^122^ Another cross‐species study identifies miR‐125b differential expression in the axolotl and the rat following spinal cord transections.^123^ Inhibiting miR‐125b in axolotls to a level similar to that in rats leads to axonal regenerative failure and deposition of fibrin (resembling glial scars observed in nonregenerative organisms).^123^

miRNAs are also pivotal in cardiac regeneration. The miR‐15 family contributes to the postnatal mitotic arrest responsible for regenerative decline throughout development.^124^, ^125^ Overexpressing miR‐195 (a member of the miR‐15 family) in neonatal mice hearts causes cell cycle arrest and prevents cardiac regeneration post‐myocardial infarction. Inhibiting miR‐195 in adult hearts, on the other hand, increases cardiomyocyte proliferation and improves cardiac function. In both cases, miR‐195 may directly target the mRNA *Chek1*, which encodes for a protein kinase promoting the G2‐M phase cell cycle transition.^124^

A more recent study shows that miR‐128 also plays a role in regulating cell cycle arrest in mice postnatally.^126^ Cardiac‐specific miR‐128 overexpression impairs cardiomyocyte proliferation and regeneration following apical resection in neonatal mice. miR‐128 deletion instead extends the cardiomyocyte proliferative window by increasing *Suz12* expression, which then suppresses *p27* expression and activates positive cell cycle regulators.^126^

miRNAs not only temper cardiomyocyte cell cycle activity, but also stimulate cell cycle re‐entry and promote cardiac regeneration. A screen for miRNAs upregulating DNA synthesis and cytokinesis in neonatal mice and rat cardiomyocytes reveals two miRNAs: hsa‐miR‐590 and hsa‐miR‐199a. These miRNAs increase both neonatal and adult cardiomyocyte proliferation in vivo and can also reduce infarct size in adult mice.^127^

Another cross‐species study characterizes two miRNA families as critical regulators of adult zebrafish heart regeneration.^128^ Cardiomyocytes within uninjured zebrafish hearts demonstrate high levels of miR‐99/100 and Let‐7a/c expression, but significantly downregulate them post‐injury. This allows *fntβ* and *smarca5* upregulation and consequent cardiomyocyte dedifferentiation and proliferation. This miRNA program is present but dormant in mammals. The inability to endogenously downregulate these miRNAs following an injury correlates with low mammalian cardiac regenerative capacity. Guided manipulation with anti‐miRs can lead to significantly reduced fibrotic scarring and enhanced cardiomyocyte dedifferentiation and proliferation in mice.^128^

## 3 POTENTIAL EXPLANATIONS FOR DIFFERENCES IN REGENERATIVE CAPACITIES AMONG VERTEBRATES

There are several potential explanations for the widely varied regenerative capacities observed across phylogeny and among vertebrates. Some of these theories include the evolutionary gain or loss of regeneration‐specific genes and molecular machinery, oxygenation and increase in oxidative damage, and the development of a more advanced immune system. Here, we will focus on the last two theories.

### 3.1 Acquisition of endothermy

Simpler vertebrates, such as fish and amphibians, are generally ectotherms, while more complex vertebrates, such as mammals and birds, are endotherms. Thyroid hormones are key regulators of energy metabolism and thermogenesis and also drive the evolutionary ectotherm‐to‐endotherm transition.^129^, ^130^ Regarding ontogenic development, a sharp postnatal increase in serum thyroid hormone levels coincides with the loss of cardiac regenerative capacity in mice.^74^, ^131^ Neonatal mice and other ectotherms—including zebrafish, axolotls and newts—have higher diploid cardiomyocyte content (indicative of higher cardiac regenerative potential) than their adult or endothermic counterparts.^131^ This trend also inversely correlates with standard metabolic rate, serum thyroid hormone levels, and body temperature. In other words, ectothermic animals demonstrate higher cardiac regenerative potential.^131^

Genetic inhibition of functional thyroid hormone receptor alpha in the heart directly shows how thyroid hormones affect mammalian cardiac regenerative potential. In this model, postnatal and adult mice demonstrate enhanced cardiomyocyte proliferation, a higher percentage of diploid cardiomyocytes, and regeneration after injury.^131^ In contrast to this, when exposed to exogenous thyroid hormones, zebrafish, which typically demonstrate robust regeneration, experience pronounced scarring, a 45% reduction in cardiomyocyte proliferation, and increased polyploidization (more reminiscent of the nonregenerative mammalian heart). These results recapitulate an evolutionarily conserved function of thyroid hormone in regulating cardiac regeneration and suggest that the decline in cardiac regenerative potential may have been a trade‐off for endothermy acquisition.

*Xenopus laevis* (African clawed frog) serves as an excellent model for exploring the relationship between thyroid hormone and ectotherm regeneration. In this species, a transient surge in thyroid hormone drives the complete metamorphosis from a highly regenerative larva to a minimally regenerative adult.^132^, ^133^ *Xenopus* tadpoles can regenerate their hindlimbs and tail but lose this capability as metamorphosis proceeds.^134^, ^135^ In respect to spinal cord axonal regeneration, accelerating metamorphosis through pharmacological thyroid hormone treatment leads to axonal growth inhibition and paralysis.^136^ *Xenopus laevis* tadpoles can also regenerate the heart but again, not after thyroid hormone‐regulated metamorphosis.^137^, ^138^ Both thyroid hormone excess and deprivation, however, lead to impaired cardiac regeneration in the tadpole, suggesting that modulation, rather than deprivation, of thyroid hormone is required for *Xenopus* regeneration.^137^

Analogous to the regenerative dichotomy that exists between zebrafish and medaka, adult *Xenopus tropicalis* (Western clawed frog) can regenerate hearts without scarring.^139^ In addition, adult *Rana temporaria* (common frog) can partially regenerate their hearts, inducing cardiomyocyte proliferation post‐injury and demonstrating almost complete functional recovery.^140^, ^141^ Regarding the two closer *Xenopus* species, a major difference in their genomes may explain the stark difference in cardiac regenerative potential: *Xenopus laevis* and all other *Xenopus* species are polyploid, while *Xenopus tropicalis* is the single exception with a diploid genome.^142^ Although more precise analysis of their respective cardiomyocyte ploidies is needed, this intrinsic genomic difference may grant *Xenopus tropicalis* greater cardiac regenerative potential over close relatives.

Another manifestation of thyroid hormone's regeneration‐conducive effect is its blastema cell proliferation enhancement and subsequent accelerated hindlimb regeneration in *Xenopus* tadpoles.^143^ This effect, however, depends on the degree of limb differentiation when amputation occurs. If the limb is amputated through an undifferentiated or differentiating region, thyroid hormone improves regeneration. Conversely, if the limb is amputated through an already differentiated region, thyroid hormone inhibits blastema formation and promotes further differentiation instead.^144^ Altogether, these findings highlight thyroid hormone's multifaceted role in regeneration and suggest that ontogenetic regenerative decline in *Xenopus* is a much more nuanced issue. However, that regenerative potential diminishes after thyroid hormone‐induced metamorphosis remains consistent. Decoupling thyroid hormone's effects from thyroid hormone‐induced metamorphic events will help us better understand if and how a transient surge in thyroid hormone levels can directly cause regenerative loss in ectothermic animals like the *Xenopus*.

### 3.2 Development of an advanced immune response

Similar to changes in thyroid hormone levels across phylogeny and ontogeny, the development of a more complex and advanced immune system parallels the regenerative decline observed across vertebrate species and within an animal's development.^145^, ^146^ *Xenopus* have simpler immune systems during highly regenerative larval stages but develop a more advanced immune system akin to that of mammals after metamorphosis.^147^ As adults, *Xenopus* are protected from viruses that kill axolotls (*Ambystoma mexicanum*). In turn, *Xenopus* lose regenerative capacity that even sexually mature but morphologically larval axolotls retain.^148^, ^149^ This may be in part due to the increase in pro‐inflammatory immune cells and cytokines that accompany an adaptive immune response. Although unclear mechanistically, this different immune response is observed during the refractory period when *Xenopus* larval tails fail to regenerate. At this time, there is a chronic immune response accompanied by a delay in local inflammation resolution and T cell maturation.^150^ Immunosuppressants can restore regenerative capacity during this refractory period, implying a correlation between a subdued or underdeveloped immune system and regeneration. More detailed studies are needed to show this relationship is causal.

That being said, an innate immune response is necessary for blastema formation in zebrafish fin and urodele limb regeneration.^151^ Successful axolotl limb regeneration requires proper inflammation modulation and macrophage recruitment.^152^ Ablating these macrophages reduces *MMP9* and *MMP3* activation and ultimately results in scarring.^153^, ^154^ Besides salamander limb regeneration, macrophages are also necessary for zebrafish fin^155^ and spinal cord^156^ regeneration as well as neonatal mouse digit tip^157^ and cardiac^158^ regeneration.

Within mammalian regeneration, neonatal mice lose their high regenerative capacity throughout development with concomitant immune system maturation.^146^, ^159^ Although pro‐regenerative macrophages with properties similar to those present in zebrafish and urodeles are found in neonatal mice hearts, they are progressively replaced with bone marrow‐derived macrophages which hinder regeneration.^160^, ^161^ As such, immunosuppression in neonatal mice inhibits cardiac regeneration, with macrophage‐secreted IL‐6 essential to initiate cardiomyocyte proliferation.^162^ To further illustrate, IL‐10 is another anti‐inflammatory cytokine which regulates the neonatal inflammatory response. After dermal injury, IL‐10 deletion in neonates results in scar tissue formation, and overexpression in postnatal mice allows for scarless wound healing.^163^, ^164^, ^165^ Besides skin wounds, IL‐10 can also improve cardiac function after myocardial infarction.^166^, ^167^, ^168^ Although present in adult mice, IL‐10 may not be upregulated enough following an injury, yielding a more pro‐inflammatory response that accelerates wound healing via fibrosis instead.

In addition to innate immunity, jawed fish possess adaptive immunity, albeit less evolved and diversified than mammals.^169^, ^170^, ^171^ Having a conserved adaptive immune system coupled with genetic tractability, zebrafish are an increasingly popular model to uncover ancestral pro‐regenerative immune signals and pathways that may have been lost or modified in mammals. In established models of spinal cord, heart, and retina regeneration, zebrafish regulatory T (T_reg_) cells modulate inflammation and promote precursor cell proliferation by secreting organ‐specific regenerative factors.^172^ Mature mouse T_reg_ cells can also decrease inflammation and promote cell differentiation and proliferation but only in regeneration‐competent tissues.^173^, ^174^, ^175^ The critical role of zebrafish T_reg_ cells in tissues normally regeneration‐incompetent in mammals has alluded to possible evolutionary pressures that limited mammalian T_reg_ cell pro‐regenerative capacity and/or altered the microenvironments in nonregenerative tissues so that they no longer stimulate T_reg_ cell pro‐regenerative activity.

Experiments in T cell‐deficient neonatal mice show that the latter hypothesis is more likely. T_reg_ cells are necessary for neonatal cardiac regeneration.^176^ Not only are T_reg_ cells from neonates pro‐regenerative, those from adult mice can also induce cardiomyocyte proliferation and regeneration in nonregenerative T cell‐deficient neonatal mice.^176^ Adoptive T_reg_ cell transfer to adult mice hearts can improve cardiac function and ameliorate adverse ventricular remodeling after injury.^177^ This transfer, however, is not sufficient for regeneration like in mutant neonates. These results indicate that the regenerative decline that accompanies mammalian immune system development is at least not due to T_reg_ cell maturation. The decline may partially be explained by an increasing pro‐fibrotic CD4^+^ T cell population but more studies are needed to annotate the changes in immune cell populations that negatively regulate regeneration.^178^

In addition to neonates, the African spiny mouse (*Acomys kempi* and *Acomys percivali*) is a unique and exceptional mammalian model capable of scarless wound healing. *Acomys* can regenerate full‐thickness skin injuries and ear holes even as adults.^179^ This trait, likely to have evolved as a defense mechanism to escape predation, may be possible due to differences in immune profiles following injuries. For instance, pro‐inflammatory factors are downregulated and pro‐reparative factors are upregulated in the *Acomys* wound bed compared to *Mus* (standard laboratory mouse strains).^180^, ^181^ Moreover, inflammatory M1 macrophages are largely absent in healing *Acomys* tissue but abundant in scarring *Mus* tissue.^181^ Although many details have yet to be teased apart, *Acomys* provides a promising platform to study robust scarless wound healing in adult mammals. Future comparative studies with regeneration in simpler vertebrates may narrow down components essential for a pro‐regenerative immune landscape.

## 4 CONCLUDING REMARKS AND FUTURE PERSPECTIVES

Are regenerative traits gained or lost throughout evolution? Are these traits positively, negatively, or neutrally selected by natural selection? Lastly, what is the major driving force that results in divergent tissue regenerative capacities across phylogeny and ontogeny? Among many theories that attempt to address these questions, ones suggesting regeneration is an ancestral trait have garnered increasing support with recent scientific discoveries.^6^ Seeing how regenerative traits are conserved throughout phylogeny, one can argue that regeneration is positively selected for if this trait correlates with increased survival. In some species, the ability to recover from physical injuries via regeneration can help individuals successfully reach maturity and pass down genes to next generations. Evidently, most simpler vertebrates retain the ability to regenerate various whole organs and body parts throughout their lifetime. At the same time, the only whole complex organ most adult mammals can regenerate is the liver. Yet, natural selection may favor traits that improve fitness to a greater extent in some species. Thus, it is possible that other traits may be selected over regenerative traits and that regeneration is lost neutrally throughout evolution.

Across evolution, regenerative potential declines with increasing phylogenetic complexity. However, while invertebrates are considered more primitive than vertebrates, insects—arguably more evolutionarily complex than flatworms but still invertebrates—do not demonstrate the same robust regeneration. There are thus parallel trends in regeneration even within invertebrate and vertebrate species, respectively.

Furthermore, this evolutionary trend also manifests as developmental changes in some vertebrates: losing regenerative traits and simultaneously gaining other evolutionary traits like endothermy and more advanced immune systems with maturation. The inverse correlation between heart regenerative potential and endothermic adequacy is an apt example.^131^ In an evolutionary perspective, the ability to regulate body temperature likely permitted early mammals to occupy new ecological niches, allowing them to thrive in colder climates and also nocturnally.^182^ Cardiac regeneration may not have been negatively selected for in this case, but rather neutrally lost as endothermy posed greater advantages in evolution. Still, why cardiac regeneration potential had to wane alongside the acquisition of endothermy across phylogeny, and why it continues to wane throughout mammalian ontogeny, requires further study.

For deeper understanding of regenerative processes or the lack thereof, it is crucial to harness nature's diversity and conduct more comparative analyses across the animal kingdom to identify shared regenerative tropes. Although zebrafish and salamanders are excellent regeneration models, they are quite distant from mammals in the phylogenetic tree. Genomic and transcriptomic divergences make it difficult, if not impossible, to pinpoint key molecular pathways underlying regenerative potential loss in most mammalian organs and appendages. Are there mammals that retain significant regenerative potential in organs and appendages that mice and humans cannot regenerate? Such mammals would enable direct tissue gene expression and physiology comparisons to help uncover molecules and pathways controlling regeneration.

Moreover, with the rapid development of next‐generation sequencing technologies and genomic editing tools such as CRISPR/Cas9, we are experiencing the emergence of many exciting genetic models in nontraditional organisms. Rigorous genetic lineage‐tracing experiments will define the cellular origins of new tissues better than traditional cell transplant assays. In addition, genetic gain‐ and loss‐of‐function studies will help elucidate candidate genes and pathways more convincingly than some current studies relying on pharmacological reagents. Armed with recent developments in single‐cell and nucleus RNA sequencing, DNA sequencing, and chromatin state analysis technologies, scientists can continue to dissect the genetic, epigenetic, and post‐transcriptional basis of regeneration across evolution.

## AUTHOR CONTRIBUTIONS

**Guo Huang:** Conceptualization; writing‐review & editing. **Sheamin Khyeam:** Conceptualization; writing‐original draft; writing‐review & editing. **Sukjun Lee:** Writing‐review.

## CONFLICT OF INTEREST

The authors declare no conflicts of interest.

### PEER REVIEW

The peer review history for this article is available at https://publons.com/publon/10.1002/ggn2.10042.

## Supporting information

Supplementary TPR File
