# Supplementary material for: Genetic, epigenetic, and post‐transcriptional basis of divergent tissue regenerative capacities among vertebrates
Source: Adv Genet (Hoboken). 2021 May 17;2(2):e10042. doi: 10.1002/ggn2.10042 (PMC8372189; doi:10.1002/ggn2.10042)
Supplement: Supplementary file 1 — Supplementary TPR File [file GGN2-2-e10042-s001.pdf]

ADVANCED GENETICS
Open Access

Genetic, Epigenetic, and Post-Transcriptional Basis of Divergent Tissue Regenerative Capacities Among Vertebrates

Sheamin Khyeam, Sukjun Lee, and Guo N. Huang*

*Corresponding

Review timeline:

Date Submitted:	16-Sep-2020
Editorial Decision:	30-Oct-2020 Minor Revision
Revision Received:	30-Apr-2021
Editorial Decision:	01-May-2021 Accept in Principle
Revision Received:	02-May-2021
Accepted:	03-May-2021

Editor: Alison Liu

Initial Editorial Evaluation	23-Sep-2020

The authors review comprehensively the regeneration capacity reserved in organs, tissues, the appendage of some vertebrate species in evolution, and discuss the contributing genes, genetic pathways, epigenetic factors, hormone, and immune systems that their conservations. The perspective is timely and of great value for improving our deeper understanding of the regeneration mechanism and evolutionary selection

1st Peer Review	25-Sep-2020 to 22-Oct-2020

Reviewer 1: Comments to the Author

This is a review and perspective on regeneration, and mechanisms involved in regeneration across animal species. It is overall a good overview; it can be published following a few improvements that should be made:

1) each subsection lists many genes required for regeneration, but they generally do not go into what is sufficient for regeneration (what functional job any of them do).

2) the paper has a lot of lists (e.g., of genes) in the text but no tables or figures. That is a missed opportunity to help the reader remember take-away points. Useful would be 1) a table or figure of what organ/tissue is regenerative in what model species, 2) genes that are actually functional to drive regeneration (for which experimental gain-of-function data are available), 3) some schematic of the regeneration process, its various steps, and what is present/absent in which species

3) I'm surprised planaria are not mentioned even once in the text. The title doesn't say the paper is only about vertebrates. It's surprising and an omission that the champions of regeneration - planaria, which offer diverse regenerative capacity even within their group - are not discussed. This needs to be added, and the existing molecular knowledge compared to the ones discussed already.

4) "V. Future Perspectives and Concluding Remarks" - very brief, and not really deep. The remarks made there do not rely on the rest of the text at all - they could have been written exactly as-is even if the info in all the rest of the paper was completely different. Therefore, I suggest something a little more profound be added here tor the reader: having seen the information in the rest of the review, what can/should they do now? What are the big ideas/conclusions that follow from the info in the paper?

5) p. 3 - “adult mammalian species are incapable of regenerating whole complex organs or body parts and are limited to regenerating specific tissues such as the skin, liver, and skeletal muscle” - what about antlers? (the authors do cover antlers later though, but it's very brief - is that really the state of the knowledge?

6) p. 5: “The deer antler is the only complex mammalian organ that can fully regenerate and” - what about the liver? I am surprised that the liver isn’t discussed here. It should be.

Reviewer 2: Comments to the Author

Overall, the is a viable and likely valuable contribution to the regenerative biology conceptual space that should see a broad readership. It is a huge swath of literature to try and tackle in one short review, and it's heavily weighted toward the issues at the end (epigenetic control, some amount of reference to hormone, some about immune system, outlook). This first draft is likely to need some work to address some critical issues, as outlined below. A few other minor issues are also noted.

Specific comments:

Throughout the Introduction, the individual paragraphs of text seem a bit disjointed from one another, creating the impression of a cataloging operation rather than a coherent piece. The paper currently reads as, "X species has been shown to regenerate Y body parts, and Z genetic pathways are known to place some role [move on to next paragraph about a different kind of animal]." There could be several ways of improving this structure, and adding a figure and/or a table would further reinforce the commonalities between species, organs/appendages, and signaling pathways.

• The second sentence of the Introduction might give naive readers that zebrafish are special among fish in being able to regenerate: "Within vertebrate species, zebrafish and salamanders are capable of regenerating the limb, fin . . . " It is possible to write this to imply that these traits are, or are likely to be, more widespread among fish (and it's already been demonstrated in some other fish, like cavefish (fins, and hearts in the surface forms). Engaging that very-cool cavefish paper somewhere in this manuscript would also be a great addition.

• In the first paragraph about mouse digit tip regeneration, it reads, " . . . neonatal mice are capable of regenerating digit tips." This paragraph should be revised to include the fact that this also occurs in adult mice and indeed, so far is known, throughout the lifetime. The same is true for humans—even though most famously reported in Ilingworth (1974), anecdotally it was appreciated in older humans for a long time, and now there are published studies supporting that it happens well after the early teen years.

• Remarks about transdifferentiation or "lineage switching" (i.e., it not being an underlying mechanism for regeneration) should be presented in a bit more restrained way. In many species, only a handful of cell types have been analyzed with the kinds of very precise genetic tools that are required to definitively answer this question for all tissues/cell types. In other species, the experiments were even less powered to answer this question. It is true that an overall theme is emerging that extensive transdifferentiation on a broad scale is not necessary for regeneration to happen nor does it appear to occur in species investigated thus far, but more studies are needed to address how far this conclusion extends and with what granularity as well as how much transdifferentiation may be provoked and a normal outcome permitted if the starting system is perturbed (that is, what the limits of plasticity are).

• Newt denervation experiments: in the strictest sense, probably the outcome of the simply denervated limb itself should not be referred to as "phenotype." I would find another word for that.

• The section on enhancer activity in cardiac regeneration is great. One of the concepts in there deserves some elaboration and possibly some discussion in other parts of this review article: injury-responsive versus regeneration-responsive. This is a key concept that is buried 1/3 of the way through the full document. Can the authors elaborate on this earlier in the work? Can they revisit it in some of the other models presented? Why does this matter (it clearly does, but the casual reader isn't likely to come away with any more impression of its importance to the central theme than when they started reading this paper).

• There are a couple typos in "killifish."

• There is an accidental inclusion of a note between the authors (which raises a good point!) in the Xenopus thyroid hormone discussion: "Should I also include research that shows TH is necessary for regeneration limbs and spinal cord in Xenopus?" Yes, you should! The effects of thyroid hormone (and immune system) are not cut-and-dry across the board and completely settled in amphibians. Including discussion of pro-regenerative roles of this hormone system not only gives the more complete picture, but shows that this can be a nuanced issue, and it might inspire someone to investigate more in the future to understand the relationship better. Right now, the evidence presented in that section also support a model in which Xenopus regenerative tendencies diminish with age, which could theoretically be decoupled from thyroid hormone (and metamorphosis) per se. What are the strongest arguments that in frogs the underlying cause is TH per se? This goes back to important differences in correlation versus causation (and it will allow you to showcase your mouse work on this topic better because in those experiments you can actually claim causation).

• I would tread lightly with the immune question, especially because the evidence one way or the other is so very rudimentary in the amphibians. Those immunosuppressants could have a variety of off-target effects, and they are like hitting the question with a giant sledgehammer. The same is true of the seminal Godwin/Rosenthal paper on macrophage depletion in axolotl limbs. I think we can all agree that this turned out to be a major foreshadowing of the conclusions to come in other (more finely-manipulatable) systems such as mice (and even zebrafish—see my next comment). However, at present, all they can really conclude is that something that eats liposomes, and therefore can be poisoned by loading them with clodronarte, is needed for regeneration. I would try to really highlight how important that paper is while also giving the flair that, so far, the precise immune responses in amphibians remain to be teased apart. Meanwhile, these more precise questions have begun to be approached in other systems. To me, one of the best papers to get into detail about here is the zebrafish Treg paper (Hui et al., 2017) because in this work, they directly show a positive requirement for Tregs in the regeneration of three different tissues/organs.

• Next section, scarless wound healing: there is no mention of what many may argue is the premier model of this at the moment, African spiny mice. It would seem a good place to include a brief mention, even if there is not space in this review for much more.

• Overall, can the authors provide any concrete evidence from the published literature that supports a possible hypothesis of regenerative prowess being an evolved trait in certain lineages or for certain organs/appendages? If not, have these approaches not been attempted with modern tools? Or, do they favor the stance there is no solid evidence? If the latter, would it be worthwhile here to engage some of the invertebrate literature (for example, out of the Rink lab) that might support acquisition of regenerative traits over evolutionary time in some specific examples?

Reviewer 3: Comments to the Author

In this timely and informative review, Khyeam and Guo outline some key aspects of regeneration across species. They focus mostly on studies of limb, heart and spinal cord and incorporate both historical and recent work from a diverse range of model and non-model organisms including teleost fish (zebrafish, killifish), amphibians (xenopus, axolotl, newt) and mammals (mice). Overall, this review is well constructed and thorough and brings to the forefront that a complex and dynamic involvement of genetic, epigenetic and post-transcriptional regulation along with systemic regulation, such as hormonal and immune system, are at the basis of an effective regeneration. Despite the enthusiasm for the topic and the potential impact of the review, several paragraphs appear as a list/summary of scientific results without properly connecting these findings and drawing meaningful conclusions. In addition, the tone of some of the text, especially in the concluding paragraph, diminishes accessibility to a broad audience.

A few suggestions below are intended for editorial purposes or to improve the manuscript.

1. Add some interpretation of the findings described. In several cases, the published data is recapped in a list but lacks a conclusion, so that the reader is left to draw parallels or inferences.
2. The references are not uniformly formatted and some are missing in the list of references, other sentences contain the wrong or missing references. Suggest using a reference management software.
3. The lack of figures or a table makes much more difficult to follow the text. Please integrate a figure or table outlining different type of regeneration well-know in animal. This could also help the authors to cut the text in the first chapter with the possibility to use a more visual explanation of the regenerative processes. Another area where a table would be useful is in summarizing the common signaling molecules that function in different tissues or across species.
4. Page 8 "can fully regenerate their hearts 60 days post-injury with no evidence of scarring through the dedifferentiation and proliferation of existing CMs". The authors should explain better what this mean. For example, saying something crucial like that the new myocardium after proper regeneration show a functionally integrated tissue at the site of the initial damage and that it can contribute again to the contractility of the ventricle.
5. Page 8 "Significant molecular pathways that mediate cardiac regeneration in neonatal mice include Hippo, neuregulin, FGF, and thyroid hormone signaling, which in one way or another, lead to changes in cardiomyocyte cell cycle activity". Please specify what "which in one way or another" means.
6. Page 10 "These genes encode regulators of the cell cycle (ccna2, ccnb1, ccnb2, ccnb3, cdc2, tk1, ube2c, uhrf1), chromosome condensation and DNA repair (smc2, smc4, ncpag, c16orf75, pcna)…". The authors should revise carefully those classifications of genes. uhrf1 is not a direct regulator of cell cycle but it is involved during DNA replication in maintenance of DNA methylation pattern cooperating with dnmt1. Also, tk1 and ube2c are not direct regulators of cell cycle. Similarly, pcna is not primarily associated with chromosome condensation and DNA repair having instead a crucial role in DNA replication fork.

7. Page 18 “Should I also include research that shows TH is necessary for regeneration of limbs and spinal cord in Xenopus?”. Authors should please remove their own drafting comment from the final text. Also, if such mentioned research is present, it should be state by the authors that contradictory research is present about thyroid hormone role in regeneration processes.
8. Page 20 “Altogether, it seems that the adult mammalian immune response is biased toward accelerated wound healing via fibrosis over regenerative healing as this presents a greater evolutionary advantage.” Please clarify how an ‘accelerated wound healing via fibrosis’ represents an evolutionary advantage.
9. Minor typos and errors are present in the text. Those are some I found that need to be fixed: Beta symbol is missing from TGF□ in page 7; Sehring, Jahn, and Weidinger, 2016 citation in page 7 is missing from the bibliography; In page 10 Oncostatin M gene is reported as Oncostatic M.

1st Editorial Decision	03-Sep-2020

Reviewer #1 recommends Minor Revision
Reviewer #2 recommends Minor Revision
Reviewer #3 recommends Minor Revision

Editor’s understanding of the reviews
Reviewers are all excellent, providing thoughtful and constructive critiques, and great suggestions to improve the manuscript. Authors should address all their concerns. Here are the recommendations based on reviews with emphasis and additional suggestions:

Editor Comments to Author:
The authors review comprehensively the regeneration capacity reserved in organs, tissues, the appendage of some vertebrate species in evolution, and discuss the contributing genes, genetic pathways, epigenetic factors, hormone, and immune systems that their conservations. The perspective is timely and of great value for improving our deeper understanding of the regeneration mechanism and evolutionary selection. Reviewers are all excellent, providing thoughtful and constructive critiques, and great suggestions to improve the manuscript. Authors should address all their concerns. Here are the recommendations based on reviews with emphasis and additional suggestions:

1. Title should reflect the content – since you limit your discussion mainly to vertebrates, you might want to include “in vertebrates” in the title to emphasize it. Under this title, you may still discuss the invertebrate regeneration that is conserved or relevant evolutionarily.

2. Please include a table with different types of regeneration well-known in animal tissues, organs, appendage, and developmental stages. It would be even better if you can also include a schematic diagram marking these species and organs on a phylogenetic or evolutionary tree with approximate time periods. In addition, you might consider marking the species that hormonal and immune system appeared and might affect the regeneration process. Adding a figure or schematic diagram can be very helpful to readers if you highlight the steps of regenerations and variations among species and organs.

3. Please use a table to summarize genes and pathways and their function in regeneration (decisive roles or not) and categorize them into genetic, epigenetic and post-transcriptional regulation. Are these factors affected by the hormones and the immune system? And additional pathways that are regulated specifically by these systems associated? To reinforce this table, you might also want to add a figure to show conserved signaling molecules that function in different tissues or across species, stressing the commonalities between species, organs/appendages, and signaling pathways during evolution.

4. Please discuss thyroid hormone in other species besides mice, emphasizing its evolutionary role in regeneration. Can non-scar regeneration be induced?

5. You might want to restructure the paragraphs with logic connections, and try to interpret the findings - what are the conclusions and new ideas based on this information reviewed?

6. The last session of the Perspective is weak and is not connected with what has been reviewed. Using all the pieces of evidence reviewed, please try to elaborate on the potential driving force for the conservation of regeneration capacity (you may include invertebrate). Natural selection is in general driven by better reproduction and survival for species, and there are often trade-offs for reserving or abandoning a function. Also, what are new challenges to understand the regeneration and apply the knowledge to medicine? What are future approaches and methods that can better tackle these challenges? Particularly, please suggest modern tools can be used to identify conserved components or key components.

Author’s Response to 1st Review	30-Apr-2021

Editor's Comments

The authors review comprehensively the regeneration capacity reserved in organs, tissues, the appendage of some vertebrate species in evolution, and discuss the contributing genes, genetic pathways, epigenetic factors, hormone, and immune systems that their conservations. The perspective is timely and of great value for improving our deeper understanding of the regeneration mechanism and evolutionary selection.

1. Title should reflect the content – since you limit your discussion mainly to vertebrates, you might want to include "in vertebrates" in the title to emphasize it. Under this title, you may still discuss the invertebrate regeneration that is conserved or relevant evolutionarily.

Response: We have changed the title to better reflect the content. Thank you for your suggestion.

2. Please include a table with different types of regeneration well-known in animal tissues, organs, appendage, and developmental stages. It would be even better if you can also include a schematic diagram marking these species and organs on a phylogenetic or evolutionary tree with approximate time periods. In addition, you might consider marking the species that hormonal and immune system appeared and might affect the regeneration process. Adding a figure or schematic diagram can be very helpful to readers if you highlight the steps of regenerations and variations among species and organs.

Response: We have included a phylogenetic tree and schematic diagram of the varying degrees of appendage, heart, and spinal cord regeneration throughout development in species discussed in the paper. We have also included approximate time periods and denoted the trends of increasing thyroid hormone levels and immune system complexity across the phylogenetic tree (p. 50).

3. Please use a table to summarize genes and pathways and their function in regeneration (decisive roles or not) and categorize them into genetic, epigenetic and post-transcriptional regulation. Are these factors affected by the hormones and the immune system? And additional pathways that are regulated specifically by these systems associated? To reinforce this table, you might also want to add a figure to show conserved signaling molecules that function in different tissues or across species, stressing the commonalities between species, organs/appendages, and signaling pathways during evolution.

Response: We have included a table summarizing essential genetic, epigenetic, and post-transcriptional factors in appendage, heart, and spinal cord regeneration, emphasizing commonalities between species if mentioned (p. 51).

4. Please discuss thyroid hormone in other species besides mice, emphasizing its evolutionary role in regeneration. Can non-scar regeneration be induced?

Response: We have further discussed the inverse relationship between thyroid hormone levels and cardiac regenerative potential observed across vertebrate species, including more details on the role of thyroid hormone specifically in zebrafish and mouse cardiac regeneration. We have also elaborated on the conflicting effects of thyroid hormone on the regenerative capacity in different tissues observed in African clawed frogs (p. 17-19).

5. You might want to restructure the paragraphs with logic connections, and try to interpret the findings - what are the conclusions and new ideas based on this information reviewed?

Response: We have revised the overall structure of the paragraphs and also added more logical connections to help with the flow and message of the paper.

6. The last session of the Perspective is weak and is not connected with what has been reviewed. Using all the pieces of evidence reviewed, please try to elaborate on the potential driving force for the conservation of regeneration capacity (you may include invertebrate). Natural selection is in general driven by better reproduction and survival for species, and there are often trade-offs for reserving or abandoning a function. Also, what are new challenges to understand the regeneration and apply the knowledge to medicine? What are future approaches and methods that can better tackle these challenges? Particularly, please suggest modern tools can be used to identify conserved components or key components.

Response: In the last section, we have revisited the question of whether regeneration is ancestral or adaptive. We argue that it is an ancestral trait that is not positively or negatively selected for but rather lost neutrally throughout evolution and also summarize evolutionary trends of regeneration across phylogeny and ontogeny. We also include

our perspectives on the challenges and opportunities in regenerative biology and medicine, as well as point out future directions using new organisms and technologies (p. 23-24).

Reviewer Comments

Reviewer #1: Comments to the Author
This is a review and perspective on regeneration, and mechanisms involved in regeneration across animal species. It is overall a good overview; it can be published following a few improvements that should be made:

1. Each subsection lists many genes required for regeneration, but they generally do not go into what is sufficient for regeneration (what functional job any of them do).s

Response: We have removed many lists and emphasized key genes that are necessary for regeneration.

s*2. The paper has a lot of lists (e.g., of genes) in the text but no tables or figures. That is a missed opportunity to help the reader remember take-away points. Useful would be 1) a table or figure of what organ/tissue is regenerative in what model species, 2) genes that are actually functional to drive regeneration (for which experimental gain-of-function data are available), 3) some schematic of the regeneration process, its various steps, and what is present/absent in which species*

Response: We have included one figure and one table to summarize the information presented: (1) a phylogenetic tree and schematic diagram of the varying degrees of appendage, heart, and spinal cord regeneration across development in species discussed and (2) a table summarizing essential genetic, epigenetic, and post-transcriptional factors in appendage, heart, and spinal cord regeneration, emphasizing commonalities between species if mentioned (p. 50-51).

3. I'm surprised planaria are not mentioned even once in the text. The title doesn't say the paper is only about vertebrates. It's surprising and an omission that the champions of regeneration - planaria, which offer diverse regenerative capacity even within their group - are not discussed. This needs to be added, and the existing molecular knowledge compared to the ones discussed already.

Response: We apologize for not making it clear that we were focusing on vertebrate regeneration. We have changed the title to reflect that. We have also included a section introducing planarian regeneration (p. 4).

4. "V. Future Perspectives and Concluding Remarks" - very brief, and not really deep. The remarks made there do not rely on the rest of the text at all - they could have been written exactly as-is even if the info in all the rest of the paper was completely different. Therefore, I suggest something a little more profound be added here tor the reader: having seen the information in the rest of the review, what can/should they do now? What are the big ideas/conclusions that follow from the info in the paper?

Response: We added discussion on the evolutionary trends of regeneration and tried to tell a narrative where the varied regenerative capacities observed in the animal kingdom may not necessarily be due to positive or negative selection; rather, regenerative traits are neutrally lost throughout evolution with the development and/or acquisition of other more beneficial evolutionary traits such as endothermy. We also include our perspectives on the challenges and opportunities in regenerative biology and medicine, as well as point out future directions using new organisms and technologies (p. 23-24).

5. p. 3 - "adult mammalian species are incapable of regenerating whole complex organs or body parts and are limited to regenerating specific tissues such as the skin, liver, and skeletal muscle" - what about antlers? (the authors do cover antlers later though, but it's very brief - is that really the state of the knowledge?

Response: We have changed the wording to: "adult mammalian species are largely limited to regenerating specific tissues such as the skin, liver, and skeletal muscle and not body parts such as the heart, spinal cord, and limbs" to be more precise (p. 3). We have also further elaborated on antler regeneration and included discussion on antlers in the section of nerve dependence (p. 5, 11).

6. p. 5: "The deer antler is the only complex mammalian organ that can fully regenerate and" - what about the liver? I am surprised that the liver isn't discussed here. It should be.

Response: We have changed the wording to: "The deer antler is the only mammalian *appendage* that can fully regenerate" (p. 5). We greatly appreciate the suggestion and have included a section on liver regeneration to highlight how it is present across vertebrate species, including mammals (p. 9).

Reviewer #2: Comments to the Author
Overall, the is a viable and likely valuable contribution to the regenerative biology conceptual space that should see a broad readership. It is a huge swath of literature to try and tackle in one short review, and it's heavily weighted toward the issues at the end (epigenetic control, some amount of reference to hormone, some about immune system, outlook). This first draft is likely to need some work to address some critical issues, as outlined below. A few other minor issues are also noted.

Specific comments:

1. Throughout the Introduction, the individual paragraphs of text seem a bit disjointed from one another, creating the impression of a cataloging operation rather than a coherent piece. The paper currently reads as, "X species has been shown to regenerate Y body parts, and Z genetic pathways are known to place some role [move on to next paragraph about a different kind of animal]." There could be several ways of improving this structure, and adding a figure and/or a table would further reinforce the commonalities between species, organs/appendages, and signaling pathways.

Response: We have revised the structure to help improve the flow of the first section and also added two figures: (1) a phylogenetic tree and schematic diagram of the varying degrees of appendage, heart, and spinal cord regeneration across development in species discussed and (2) a table summarizing essential genetic, epigenetic, and post-transcriptional factors in appendage, heart, and spinal cord regeneration, emphasizing commonalities between species if mentioned (p. 50-51).

2. The second sentence of the Introduction might give naive readers that zebrafish are special among fish in being able to regenerate: "Within vertebrate species, zebrafish and salamanders are capable of regenerating the limb, fin . . . " It is possible to write this to imply that these traits are, or are likely to be, more widespread among fish (and it's already been demonstrated in some other fish, like cavefish (fins, and hearts in the surface forms). Engaging that very-cool cavefish paper somewhere in this manuscript would also be a great addition.

Response: We have re-worded the sentence so that zebrafish is listed as an example instead: "Within vertebrate species, phylogenetically primitive organisms such as fish (with zebrafish being a prominent example) and urodele amphibians…" (p. 3). We thank the reviewer for the additional suggestion and have added discussion about the cavefish paper in the cardiac regeneration section (p. 8).

3. In the first paragraph about mouse digit tip regeneration, it reads, " . . . neonatal mice are capable of regenerating digit tips." This paragraph should be revised to include the fact that this also occurs in adult mice and indeed, so far is known, throughout the lifetime. The same is true for humans—even though most famously reported in Ilingworth (1974), anecdotally it was appreciated in older humans for a long time, and now there are published studies supporting that it happens well after the early teen years.

Response: We have revised the paragraph to state that adult mice and humans (citing the Illingworth paper) can regenerate the digit tip as well (p. 5).

4. Remarks about transdifferentiation or "lineage switching" (i.e., it not being an underlying mechanism for regeneration) should be presented in a bit more restrained way. In many species, only a handful of cell types have been analyzed with the kinds of very precise genetic tools that are required to definitively answer this question for all tissues/cell types. In other species, the experiments were even less powered to answer this question. It is true that an overall theme is emerging that extensive transdifferentiation on a broad scale is not necessary for regeneration to happen nor does it appear to occur in species investigated thus far, but more studies are needed to address how far this conclusion extends and with what granularity as well as how much transdifferentiation may be provoked and a normal outcome permitted if the starting system is perturbed (that is, what the limits of plasticity are).

Response: Thank you–we agree with the reviewer's suggestion and have carefully reworded sentences discussing the "absence" of lineage switching, alluding to the limitations of current studies (p. 4, 6). We have also included an example where there is evidence of lineage switching in the axolotl spinal cord regeneration model (p. 6) and emphasize the need for more rigorous genetic lineage-tracing experiments in the conclusion (p. 24).

5. Newt denervation experiments: in the strictest sense, probably the outcome of the simply denervated limb itself should not be referred to as "phenotype." I would find another word for that.

Response: We have removed the description that it is a "phenotype" and have simply described the phenomenon as "sufficient to induce distal structure regeneration" (p. 10).

6. The section on enhancer activity in cardiac regeneration is great. One of the concepts in there deserves some elaboration and possibly some discussion in other parts of this review article: injury-responsive versus regeneration-responsive. This is a key concept that is buried 1/3 of the way through the full document. Can the authors elaborate on this earlier in the work? Can they revisit it in some of the other models presented? Why does this matter (it clearly does, but the casual reader isn't likely to come away with any more impression of its importance to the central theme than when they started reading this paper).

Response: We have introduced this concept in the introduction as a question that still warrants further investigation (p. 3). Due to structural purposes, we have decided to keep most of the discussion in the later section (p. 13).

7. There are a couple typos in "killifish."

Response: We have addressed these typos (p. 13).

8. There is an accidental inclusion of a note between the authors (which raises a good point!) in the Xenopus thyroid hormone discussion: "Should I also include research that shows TH is necessary for regeneration limbs and spinal cord in Xenopus?" Yes, you should! The effects of thyroid hormone (and immune system) are not cut-and-dry across the board and completely settled in amphibians. Including discussion of pro-regenerative roles of this hormone system not only gives the more complete picture, but shows that this can be a nuanced issue, and it might inspire someone to investigate more in the future to understand the relationship better. Right now, the evidence presented in that section also support a model in which Xenopus regenerative tendencies diminish with age, which could theoretically be decoupled from thyroid hormone (and metamorphosis) per se. What are the strongest arguments that in frogs the underlying cause is TH per se? This goes back to important differences in correlation versus causation (and it will allow you to showcase your mouse work on this topic better because in those experiments you can actually claim causation).

Response: We thank the reviewer for the kind and helpful suggestion regarding our accidental inclusion. We have included examples and discussion of the pro-regenerative roles of thyroid hormone in frog heart and limb regeneration and have also emphasized the need to decouple the effects of thyroid hormone and metamorphosis in order to fully understand its role in *Xenopus* regeneration (p. 18-19).

9. I would tread lightly with the immune question, especially because the evidence one way or the other is so very rudimentary in the amphibians.   Those immunosuppressants could have a variety of off-target effects, and they are like hitting the question with a giant sledgehammer. The same is true of the seminal Godwin/Rosenthal paper on macrophage depletion in axolotl limbs.   I think we can all agree that this turned out to be a major foreshadowing of the conclusions to come in other (more finely-manipulatable) systems such as mice (and even zebrafish—see my next comment).   However, at present, all they can really conclude is that something that eats liposomes, and therefore can be poisoned by loading them with clodronarte, is needed for regeneration. I would try to really highlight how important that paper is while also giving the flair that, so far, the precise immune responses in amphibians remain to be teased apart.   Meanwhile, these more precise questions have begun to be approached in other systems.   To me, one of the best papers to get into detail about here is the zebrafish Treg paper (Hui et al., 2017) because in this work, they directly show a positive requirement for Tregs in the regeneration of three different tissues/organs.

Response: We thank the reviewer for the thorough suggestion. We have tried to emphasize the rudimentary nature of the evidence presented in amphibians and have also added discussion on the zebrafish Treg paper. To incorporate another example with concrete genetic evidence, we further discuss a positive requirement for Tregs in neonatal mice heart regeneration as well (p. 20-22).

10. Next section, scarless wound healing: there is no mention of what many may argue is the premier model of this at the moment, African spiny mice.   It would seem a good place to include a brief mention, even if there is not space in this review for much more.

Response: Thank you for the suggestion. We have included discussion on scarless wound healing observed in African spiny mice as well as how their immune composition plays a role during this process (p. 22).

11. Overall, can the authors provide any concrete evidence from the published literature that supports a possible hypothesis of regenerative prowess being an evolved trait in certain lineages or for certain organs/appendages? If not, have these approaches not been attempted with modern tools? Or, do they favor the stance there is no solid evidence? If the latter, would it be worthwhile here to engage some of the invertebrate literature (for example, out of the Rink lab) that might support acquisition of regenerative traits over evolutionary time in some specific examples?

> Response: We apologize if the original hypothesis set forth was not clear in the first draft. Although we acknowledge the hypothesis of the acquisition of regenerative traits briefly in the introduction, we try to argue that regenerative prowess is more of an ancestral trait that is gradually lost throughout evolution. We do greatly appreciate the suggestion though and added some discussion on invertebrates as well as elaborated on the original hypothesis in the last section (p. 23-24).

Reviewer #3: Comments to the Author

In this timely and informative review, Khyeam and Guo outline some key aspects of regeneration across species. They focus mostly on studies of limb, heart and spinal cord and incorporate both historical and recent work from a diverse range of model and non-model organisms including teleost fish (zebrafish, killifish), amphibians (xenopus, axolotl, newt) and mammals (mice). Overall, this review is well constructed and thorough and brings to the forefront that a complex and dynamic involvement of genetic, epigenetic and post-transcriptional regulation along with systemic regulation, such as hormonal and immune system, are at the basis of an effective regeneration. Despite the enthusiasm for the topic and the potential impact of the review, several paragraphs appear as a list/summary of scientific results without properly connecting these findings and drawing meaningful conclusions. In addition, the tone of some of the text, especially in the concluding paragraph, diminishes accessibility to a broad audience.

A few suggestions below are intended for editorial purposes or to improve the manuscript.

1. Add some interpretation of the findings described. In several cases, the published data is recapped in a list but lacks a conclusion, so that the reader is left to draw parallels or inferences.

> Response: We have revised the text so that there are more concrete conclusions and logical reasonings behind the evidence presented.

2. The references are not uniformly formatted and some are missing in the list of references, other sentences contain the wrong or missing references. Suggest using a reference management software.

> Response: We have used a reference management software and fixed the references accordingly.

3. The lack of figures or a table makes much more difficult to follow the text. Please integrate a figure or table outlining different type of regeneration well-known in animal. This could also help the authors to cut the text in the first chapter with the possibility to use a more visual explanation of the regenerative processes. Another area where a table would be useful is in summarizing the common signaling molecules that function in different tissues or across species.

> Response: We have included one figure and one table to summarize the information presented: (1) a phylogenetic tree and schematic diagram of the varying degrees of appendage, heart, and spinal cord regeneration across development in species discussed and (2) a table summarizing essential genetic, epigenetic, and post-transcriptional factors in appendage, heart, and spinal cord regeneration, emphasizing commonalities between species if mentioned (p. 50-51).

4. Page 8 "can fully regenerate their hearts 60 days post-injury with no evidence of scarring through the dedifferentiation and proliferation of existing CMs". The authors should explain better what this mean. For example, saying something crucial like that the new myocardium after proper regeneration show a functionally integrated tissue at the site of the initial damage and that it can contribute again to the contractility of the ventricle.

> Response: We have edited the sentence to elaborate what it means to fully regenerate the heart by adding: "…with damaged tissue completely restored, structurally and functionally" (p. 8).

5. Page 8 "Significant molecular pathways that mediate cardiac regeneration in neonatal mice include Hippo, neuregulin, FGF, and thyroid hormone signaling, which in one way or another, lead to changes in cardiomyocyte cell cycle activity". Please specify what "which in one way or another" means.

Response: We have rephrased the sentence to prevent confusion: "Significant molecular pathways that mediate cardiac regeneration in neonatal mice include Hippo, neuregulin, FGF, and thyroid hormone signaling, each altering cardiomyocyte cell cycle activity." We considered specifying that these alterations can lead to either positive or negative cardiomyocyte cell cycle activity but refrained from doing so due to redundancy (p. 8-9).

6. Page 10 "These genes encode regulators of the cell cycle (ccna2, ccnb1, ccnb2, ccnb3, cdc2, tk1, ube2c, uhrf1), chromosome condensation and DNA repair (smc2, smc4, ncpag, c16orf75, pcna)…". The authors should revise carefully those classifications of genes. uhrf1 is not a direct regulator of cell cycle but it is involved during DNA replication in maintenance of DNA methylation pattern cooperating with dnmt1. Also, tk1 and ube2c are not direct regulators of cell cycle. Similarly, pcna is not primarily associated with chromosome condensation and DNA repair having instead a crucial role in DNA replication fork.

Response: We thank the reviewer for taking the time to point out the incorrect classifications of these genes. Due to space limitations and restructuring of the paper, we have decided to omit most of these genes and instead emphasize *ccna2* as it has been shown to be promote regeneration in other models as well (p. 10).

7. Page 18 "Should I also include research that shows TH is necessary for regeneration of limbs and spinal cord in Xenopus?". Authors should please remove their own drafting comment from the final text. Also, if such mentioned research is present, it should be state by the authors that contradictory research is present about thyroid hormone role in regeneration processes.

Response: We apologize for the unprofessional accidental inclusion in the text. We have included discussion on contradictory research present in literature (p. 18-19).

8. Page 20 "Altogether, it seems that the adult mammalian immune response is biased toward accelerated wound healing via fibrosis over regenerative healing as this presents a greater evolutionary advantage." Please clarify how an 'accelerated wound healing via fibrosis' represents an evolutionary advantage.

Response: We thank the reviewer for pointing this out. We originally intended to suggest that scarring after an *open* wound presents an evolutionary advantage over regeneration of the heart/spinal cord/appendage as it prevents infections which may be more likely to kill the animal before regeneration is complete but realized this does not address cases where there are no open wounds. After some careful deliberation, we have decided to remove this statement to avoid confusion.

9. Minor typos and errors are present in the text. Those are some I found that need to be fixed: Beta symbol is missing from TGFb in page 7; Sehring, Jahn, and Weidinger, 2016 citation in page 7 is missing from the bibliography; In page 10 Oncostatin M gene is reported as Oncostatic M.

Response: We have made the following changes and also carefully reviewed the text for any additional typos and errors.

2nd Editorial Decision	Editor: Myles Axton	01-May-2021

The authors have responded to and incorporated all of the editor's and reviewers' points into the revised Perspective which is now accepted in principle, subject to textual edits and formatting in keeping with journal style.

Author's Response	02-May-2021

We greatly appreciate such a prompt response and valuable advices on the manuscript. The review has now been edited to incorporated suggested changes. Please let us know if there is any question.
